# Myeloid-derived suppressor cell and macrophage exert distinct angiogenic and immunosuppressive effects in breast cancer

**DOI:** 10.18632/oncotarget.17013

**Published:** 2017-04-10

**Authors:** Zhaoxu Fang, Chengwen Wen, Xiaolan Chen, Rongping Yin, Chenglin Zhang, Xiaohua Wang, Yuhui Huang

**Affiliations:** ^1^ Cyrus Tang Hematology Center, Jiangsu Institute of Hematology, Collaborative Innovation Center of Hematology, Soochow University, Suzhou, China; ^2^ Institute of Pediatric Research, Affiliated Children's Hospital, Soochow University, Suzhou, China; ^3^ School of Nursing, Soochow University, Suzhou, China; ^4^ The First Affiliated Hospital of Soochow University/School of Nursing, Soochow University, Suzhou, China; ^5^ Key Laboratory of Stem Cells and Biomedical Materials of Jiangsu Province and Chinese Ministry of Science and Technology, Soochow University, Suzhou, China

**Keywords:** myeloid-derived suppressor cell, tumor-associated macrophage, immunosuppression, angiogenesis, immunotherapy

## Abstract

The immunosuppressive tumor microenvironment is a key obstacle to hinder a cancer immunotherapy. Myeloid-derived suppressor cells (MDSCs) have been considered as a major player in immunosuppression. In this study, we find that tumor-infiltrating MDSCs (tiMDSCs) are less immunosuppressive than tumor-associated macrophages (TAMs) in multiple murine orthotopic breast tumor models. Compared to TAMs, tiMDSCs produce higher levels of pro-inflammatory factors and lower levels of anti-inflammatory factors. Furthermore, tiMDSCs are preferentially located in hypoxic areas and are more pro-angiogenic than TAMs. Consistent with these functional disparities, a shift from tiMDSCs to TAMs is observed during the progression of breast cancer. Moreover, infiltration of tiMDSCs is also noted in distal colonization of breast cancer cells in the lung. Taken together, our findings indicate that tiMDSCs are more pro-angiogenic and promote tumor initiation, while TAMs are more immunosuppressive and facilitate tumor immune evasion. This study suggests that selectively targeting on TAMs could alleviate the immunosuppressive tumor microenvironment and potentiate cancer immunotherapy.

## INTRODUCTION

Inducing angiogenesis and evading host immune destruction are two hallmarks of cancer [1]. Tumor-infiltrating myeloid cell, including myeloid-derived suppressor cell (MDSC), tumor-associated neutrophil (TAN), and tumor-associated macrophage (TAM) plays diverse roles in tumor progression [2–6].

TAM represents a dominant myeloid cell population within many kinds of cancer lesions, and its accumulation often correlates with poor prognosis [4, 7, 8]. TAM usually has an M2-like phenotype and expresses typical markers, such as CD11b and F4/80 in murine models. M2-like TAM promotes tumor progression through multiple mechanisms, including the support of tumor angiogenesis, the induction of tumor cell invasion and migration, the promotion of extracellular matrix remodeling, and the suppression of host anti-cancer immune responses [8–12].

TAN expresses two typical neutrophil markers, CD11b and Ly-6G, in murine tumor models. TAN is involved in tumor initiation and progression via several mechanisms, including pro-angiogenesis and immunosuppression [6, 13, 14]. In a mesothelioma AB12 tumor model, depletion of Ly-6G neutrophils *in vivo* induced CD8^+^ T cell activation, indicating the immunosuppression of TAN [5].

MDSC has been identified in cancer patients and tumor-bearing mice. MDSC is a heterogeneous myeloid cell population with ability to suppress T cell activation. In tumor-bearing mice, MDSC is CD11b^+^Gr1^+^ and accumulates in the bone marrow, the spleen, and peripheral blood [15–19].

Although the phenotypes and functions of MDSC in peripheral immune organs are well defined, what are the critical roles of MDSC in the tumor microenvironment, as well as its relationship with TAN and TAM, remains not fully understood [6, 20, 21]. In this study, we characterized the immunological and angiogenic properties of these tumor-infiltrating myeloid cells in breast tumor models. Our data showed that tumor-infiltrating MDSC (tiMDSC) was less immunosuppressive, while more angiogenic, than TAM. Thus, selectively targeting TAM, rather than tiMDSC, could recondition the immunosuppressive tumor microenvironment and improve the efficacy of cancer immunotherapy.

## RESULTS

### TiMDSC and TAM are two major tumor-infiltrating myeloid cell populations in spontaneous and orthotopic breast tumors

**Figure 1:**
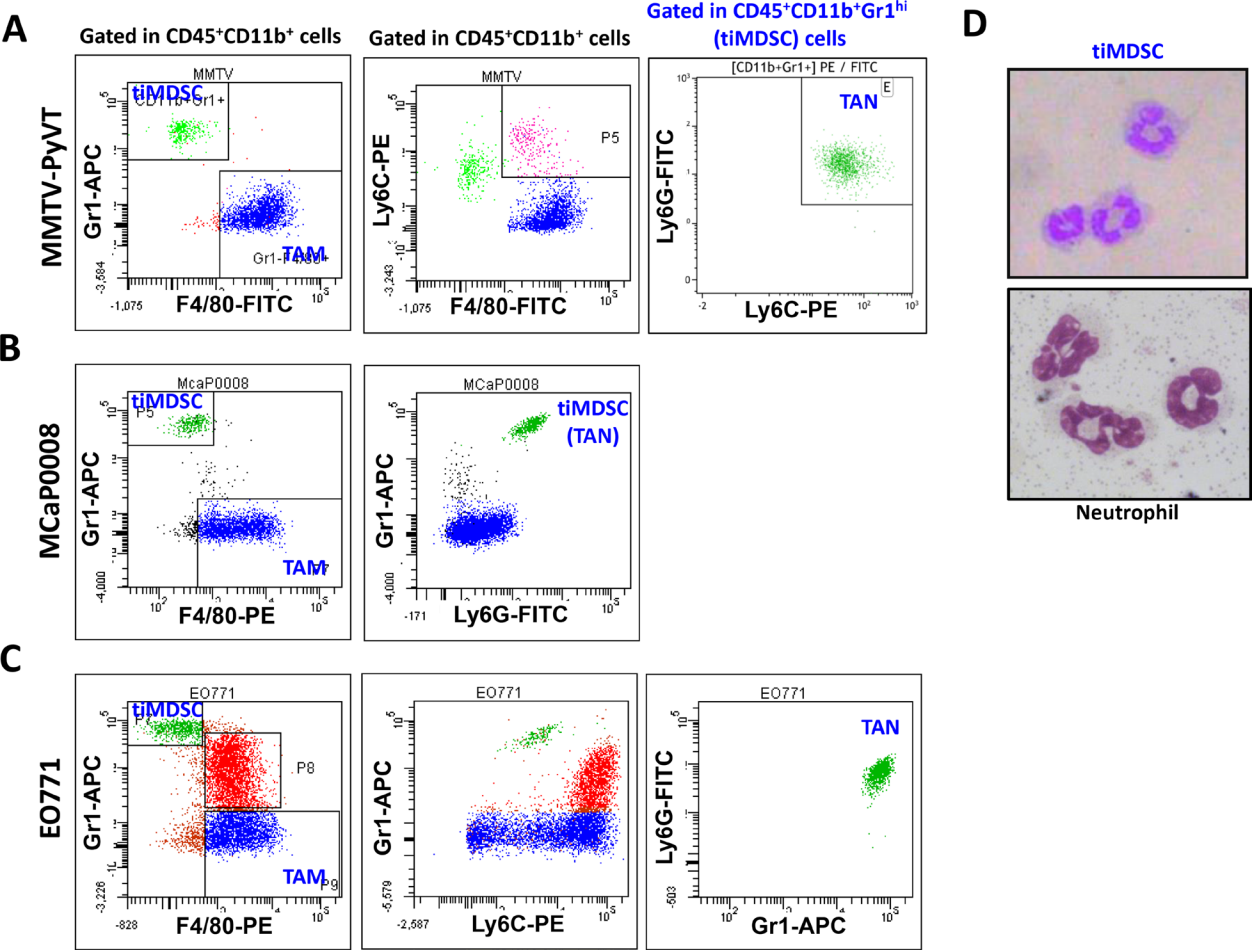
Phenotypes of tumor-infiltrating myeloid cell populations in breast tumor models Single cell suspensions were prepared from breast tumor tissues. Expression of Gr1, F4/80, Ly6G, and Ly6C were analyzed in CD45^+^CD11b^+^ cells by flow cytometry. Representative flow images were shown. (**A**) CD45^+^CD11b^+^Gr1^hi^F4/80^−^ and CD45^+^CD11b^+^Gr1^−^F4/80^+^ cells comprised two major populations in spontaneous MMTV−PyVT breast tumors. (**B**) and (**C**) There were three tumor-infiltrating myeloid cell populations in orthotopically implanted MCaP0008 and EO771 breast tumors. In all breast tumor models evaluated, the CD45^+^CD11b^+^Gr1^hi^F4/80^−^ cell population was Ly6G^+^Ly6C^low^. (**D**) Giemsa staining of cytospin preparations of tiMDSC and neutrophil. CD45^+^CD11b^+^Gr1^hi^F4/80^−^ cells (tiMDSC) were purified from MMTV-PyVT breast tumor tissues. CD45^+^CD11b^+^Ly6G^+^Ly6C^−^ cells (neutrophil) were isolated from peripheral blood. The phenotypes of tiMDSC, TAN and TAM were repeated more than 5 times.

In the peripheral immune organs, such as lymph nodes and spleen, MDSC is considered to be a major immune suppressor [2, 15, 22]. Our previous study showed that low dose anti-VEGFR2 treatment improved cancer vaccine therapy, even though tiMDSC was increased [23]. These results lead us to hypothesize that tiMDSC is not the major immune suppressor within the tumor microenvironment. To get more insights into the phenotypes of tumor-infiltrating myeloid cell populations, we established representative murine breast cancer models: spontaneously arising autochthonous mammary carcinoma (MMTV-PyVT) and orthotopic implanted breast cancers (EO771 and MCaP0008). MMTV-PyVT is a widely used murine breast cancer model that mirrors the progression of breast cancer in humans [24, 25]. In MMTV-PyVT breast tumor tissue, two major tumor-infiltrating myeloid cell populations were identified: CD45^+^CD11b^+^Gr1^hi^F4/80^−^ (Gr1^+^F4/80^−^, tiMDSC) and CD45^+^CD11b^+^Gr1^−^F4/80^+^ (Gr1^−^F4/80^+^, TAM) (Figure 1A and Supplementary Figure 1). In EO771 and MCaP0008 tumors, there were three major myeloid cell populations: CD45^+^CD11b^+^Gr1^hi^F4/80^−^ (tiMDSC), CD45^+^CD11b^+^Gr1^int/low^F4/80^int/low^, and CD45^+^CD11b^+^Gr1^−^F4/80^+^ (TAM) (Figure 1B–1C and Supplementary Figures 2–3,). In all breast tumor models tested here, CD11b^+^Gr1^hi^F4/80^−^ (tiMDSC) cells were also Ly6G^+^Ly6C^low^, an equivalent phenotype to that observed in TAN. Giemsa staining also indicated that CD11b^+^Gr1^hi^F4/80^−^ (tiMDSC) cells had typical characteristics of neutrophil (Figure 1D). Most CD45^+^CD11b^+^Gr1^int/low^F4/80^int/low^ cells were Ly6G^−^Ly6C^+^, suggesting that they are monocytic myeloid cells (Figure 1C). In the breast cancer models evaluated here, the majority of TAMs were Gr1^−^Ly6G^−^, but some of them were Ly6C^+^ (Figure 1). In EO771 cancer models, myeloid cell populations displayed very different patterns compared to the other two models tested in this study. CD45^+^CD11b^+^Gr1^int/low^F4/80^int/low^ cells were a big population, and most of them were Ly6G^−^Ly6C^+^. In addition, many TAMs also expressed Ly−6C in EO771 tumor (Figure 1C). Together, these data suggest that tiMDSC has a similar phenotype to TAN (CD11b^+^Gr1^+^Ly6G^+^Ly6C^low^F4/80^−^). TiMDSC and TAM comprise two distinct tumor-infiltrating myeloid cell populations in established breast tumors.

### TAM is more potent than tiMDSC in the suppression of T cell proliferation stimulated by anti-CD3/CD28 monoclonal antibodies

**Figure 2:**
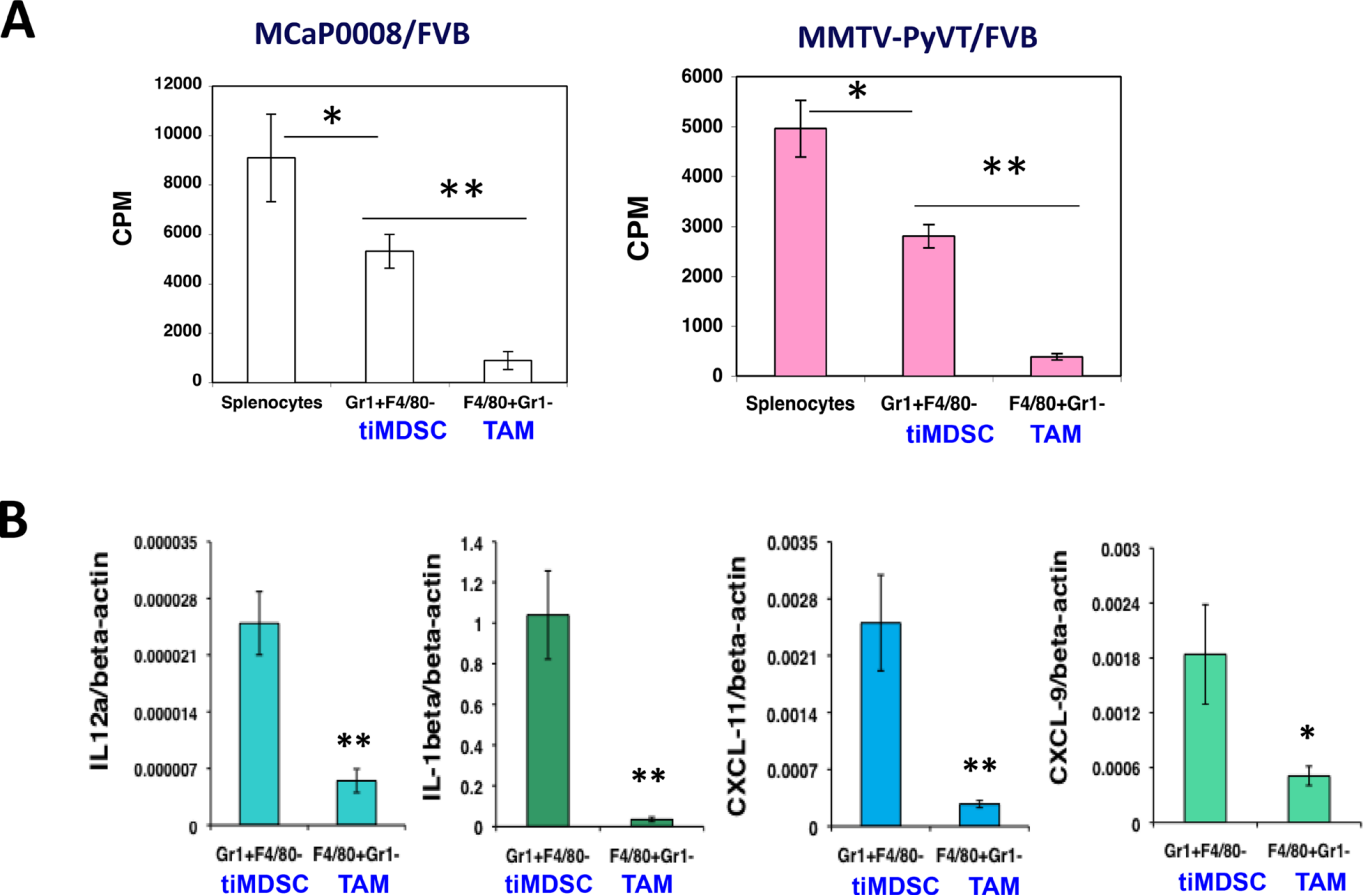
TAM is more immunosuppressive than tiMDSC in breast tumors CD11b microbead was used to enrich tumor-infiltrating myeloid cells from MCaP0008 and MMTV-PyVT breast tumors. Flow sorting was used to purify tumor-infiltrating MDSC (tiMDSC: CD45^+^CD11b^+^Gr1^hi^F4/80^−^) and TAM (CD45^+^CD11b^+^Gr1^−^F4/80^+^). TiMDSCs and TAMs (5 × 10^4^ cells) were then co-cultured with splenocytes (2 × 10^5^ cells) for 24 hrs with anti-CD3/CD28 (1/5 μg/ml), and pulsed overnight with 1 μCi of ^3^H-thymidine. Cells were harvested, and ^3^H-thymidine uptake was measured. Splenocytes (2 × 10^5^ cells) cultured in anti-CD3/CD28 (1/5 ug/ml) without myeloid cells were used as controls. (**A**) TAM inhibited T cell proliferation more potently than tiMDSC in MCaP0008 and MMTV-PyVT breast cancer models. (**B**) TiMDSC expressed higher levels of proinflammatory factors in MCaP0008 breast cancer. Data were shown as mean values ± SEM (*n* = 5–8 mice per group). Experiments were repeated three times. * denotes *P* < 0.05, ** denotes *P* < 0.01.

As tumor-infiltrating myeloid cells have been suggested to play critical roles in the immunosuppression [18, 26], but the distinct roles of tiMDSC and TAM in tumor immune evasion remain not very clear. Thus, we compared the capability of TAM and tiMDSC to suppress T cell function by co-culturing them with splenocytes at the same ratio. In both MMTV-PyVT and MCaP0008 breast cancer models, tiMDSC (Gr1^+^F4/80^−^) inhibited T cell proliferation induced by anti-CD3/CD28 monoclonal antibodies; however, TAM (Gr1^−^F4/80^+^) was much more potent than tiMDSC in suppressing T cell proliferation (Figure 2A). The data showed that TAM is more immunosuppressive than tiMDSC in breast tumors.

**Figure 3:**
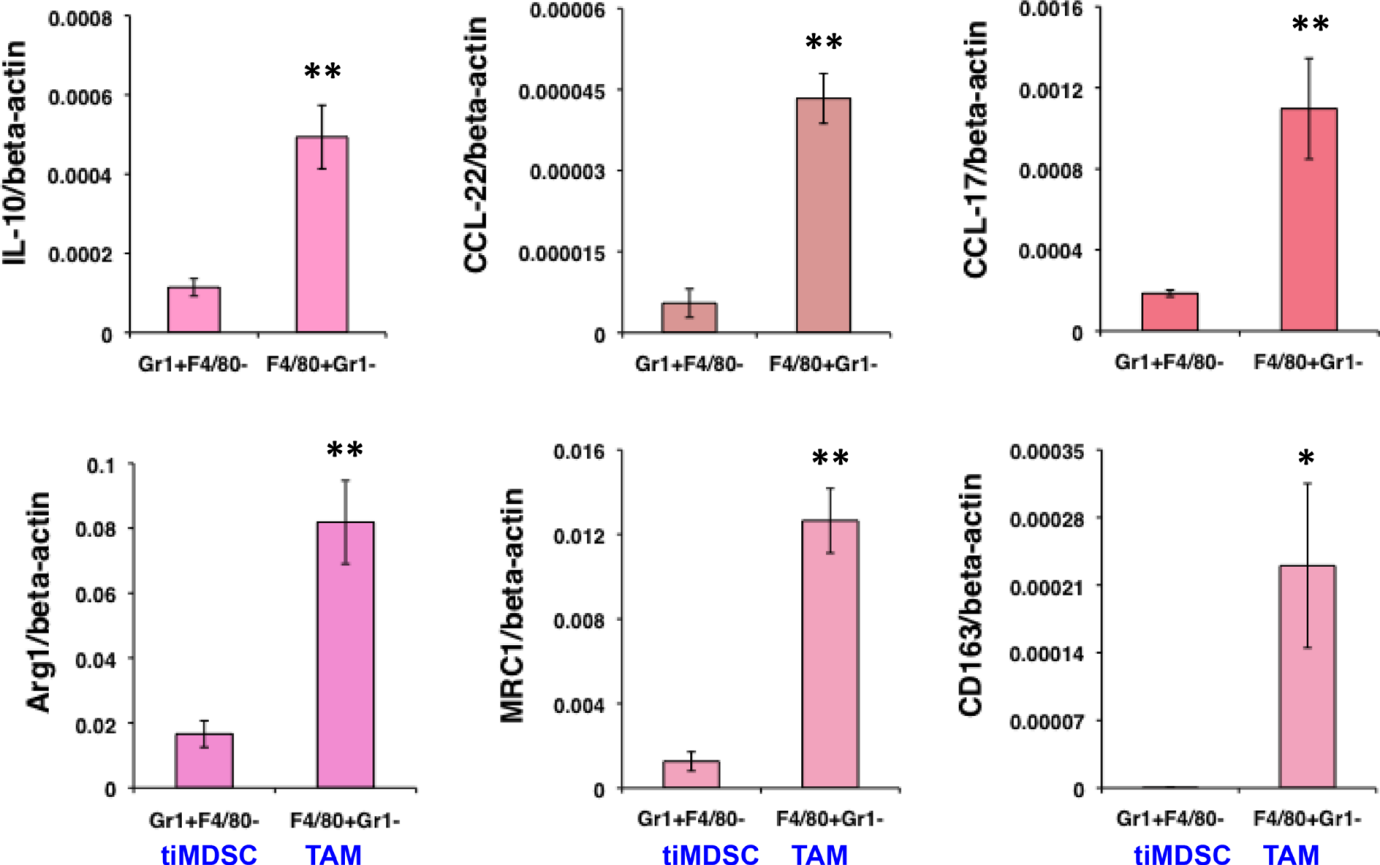
TiMDSC expresses lower levels of anti-inflammatory factors compared to those of TAM in MCaP0008 breast cancers Gene expression profiles of tiMDSC and TAM were analyzed by qRT-PCR. The experiment procedure was the same as described in Figure 2. Data were shown as mean values ± SEM (*n* = 6–8 mice per group). Experiments were repeated four times. * denotes *P* < 0.05, ** denotes *P* < 0.01.

**Figure 4:**
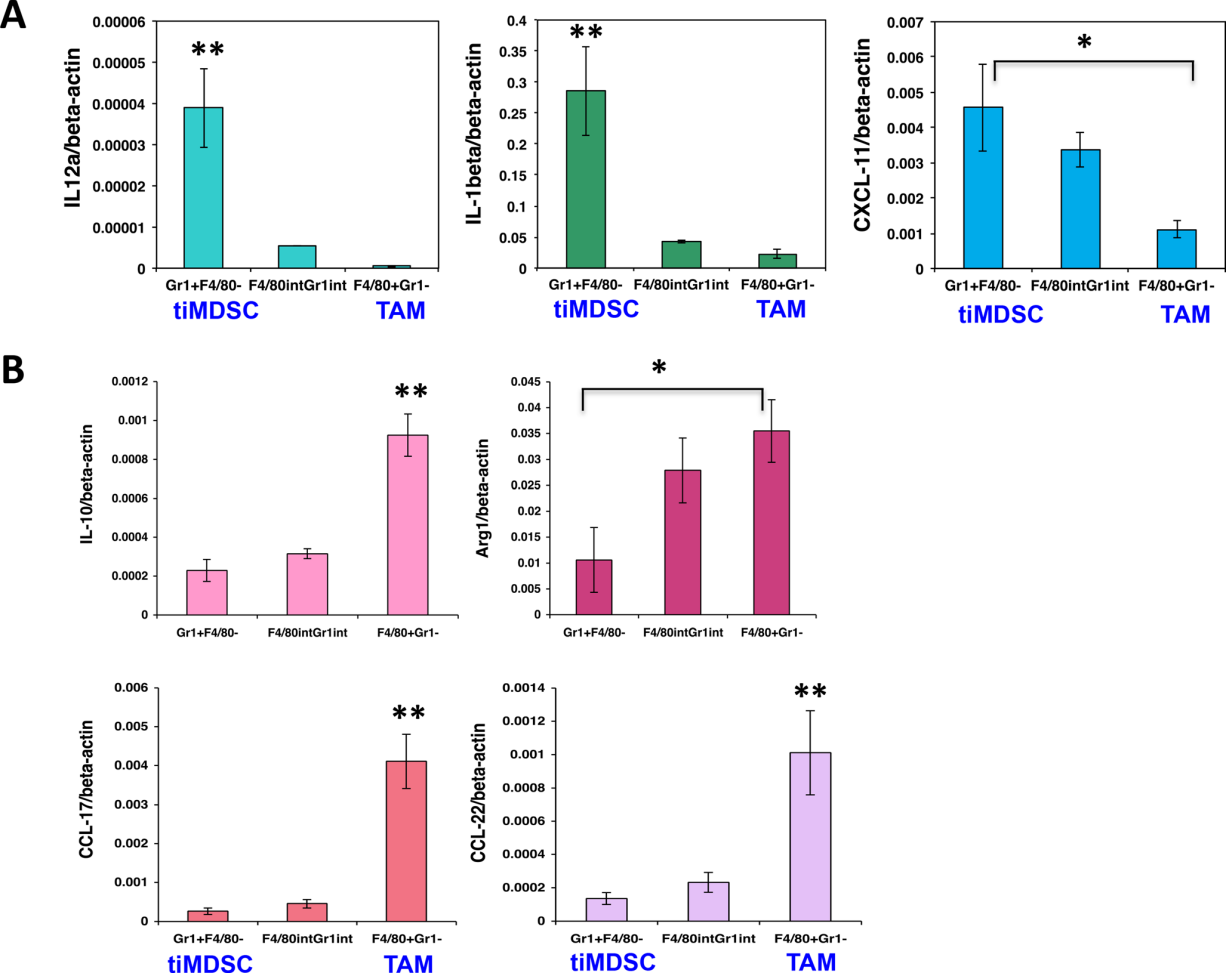
TiMDSC expresses higher levels of pro-inflammatory factors and lower levels of anti-inflammatory factors in EO771 breast tumors Gene expression profiles were analyzed by qPCR. (**A**) TiMDSC expressed higher levels of pro-inflammatory factors in EO771 breast cancer. (**B**) TAM expressed higher levels of anti-inflammatory factors in EO771 breast cancer. Data were shown as mean values ± SEM (*n* = 6–8 mice per group). Experiments were repeated three times. * denotes *P* < 0.05, **denotes *P* < 0.01.

To reveal molecular mediators involved in immune suppression, we isolated tiMDSC and TAM from MCaP0008 cancer tissues using CD11b-microbead enrichment followed by flow sorting. Then, we analyzed the cytokine/chemokine profiles of tiMDSC and TAM. TiMDSC had significantly higher levels of pro-inflammatory factors, such as *IL12α, IL-1β, CXCL9* and *CXCL10*, compared to TAM (Figure 2B). Conversely, tiMDSC had significantly lower levels of anti-inflammatory factors, including *IL10, Arg1, CCL17*, and *CCL22*, compared to TAM (Figure 3). In EO771 tumor model, we also analyzed the gene expression profiles in tiMDSC, TAM and CD45^+^CD11b^+^Gr1^int/low^F4/80^int/low^ cells. Again, tiMDSC expressed higher levels of *IL12α, IL1β, CXCL11*, while TAM had higher levels of *IL10, Arg1, CCL17* and *CCL22* (Figure 4). The gene expression profile of CD45^+^CD11b^+^Gr1^int/low^F4/80^low^ was in between those measured for tiMDSC and TAM. Together, these data suggest that TAM is more immunosuppressive than tiMDSC, consistent with functional data (Figure 2).

### TiMDSC is accumulated in non-perfused tumor areas in breast cancer

**Figure 5:**
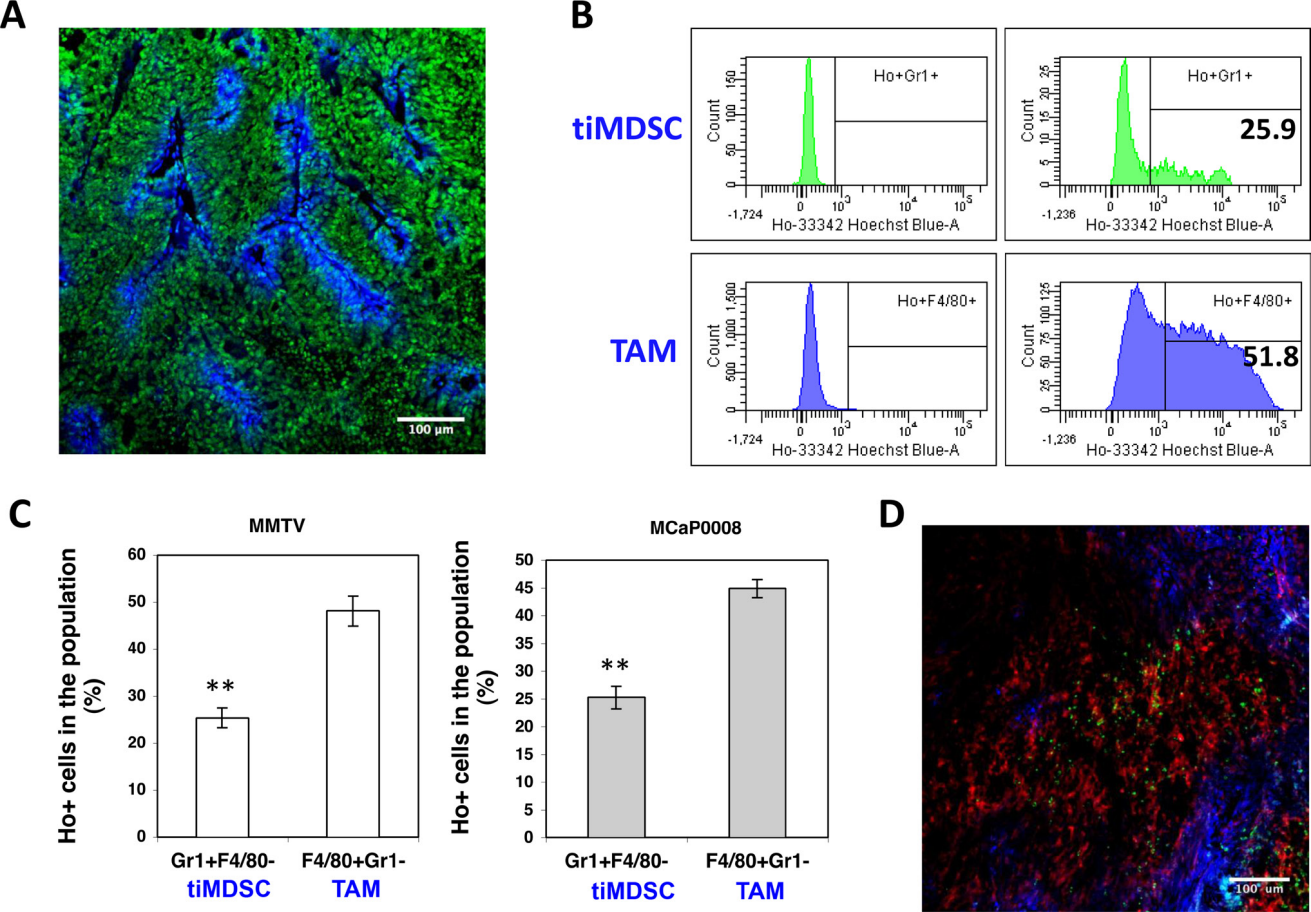
TiMDSC is preferentially localized in hypoxic/necrotic areas in breast tumor models (**A**) A representative confocal image of Hoechst 33342 perfused MCaP0008 tumor tissue. Sytox Green (Green), Hoechst 33342 (Blue). Scale bar is 100 μm. (**B**) Representative histogram of Hoechst 33342 positive tiMDSC or TAM presented in MMTV-PyVT breast cancers. Numbers indicate the percentage of Hoechst 33342 positive tiMDSCs or TAMs. (**C**) The proportion of Hoechst 33342 positive tiMDSC and TAM cells in MMTV-PyVT (*n* = 5 mice) and MCaP0008 breast cancers (*n* = 8 mice). (**D**) The distribution of tiMDSCs and TAMs in MCaP0008 tumor tissues. Green: Ly6G^+^ cells (tiMDSCs); Red: F4/80^+^ cells (TAMs); Blue: Hoechst 33342 perfused vessels. Experiments were repeated four times. Data were shown as means ± SEM.

TAM is usually considered to accumulate in the hypoxic/necrotic areas of tumor tissue [27, 28], while the distribution of tiMDSC in tumor tissue remains unclear. In order to evaluate the distribution of tiMDSC and TAM *in vivo*, we adapted an intravital Hoechst 33342 staining technique [23, 29, 30] to label myeloid cells as Hoechst 33342 positive (Ho^+^, proximal to perfused tumor vessels) and negative (Ho^−^, hypoxic/necrotic area). Then, we analyzed their distribution using flow cytometry (Figure 5A). In both MMTV-PyVT and MCaP0008 tumor models, Ho^+^tiMDSCs were approximately 25% while Ho^+^TAMs were about 50% (Figure 5B–5C). From the patterns of Hoechst fluorescence intensity histograms, both Ho^+^tiMDSCs and Ho^+^TAMs were found to be evenly distributed, indicating that both tiMDSCs and TAMs were evenly distributed around the perfused tumor vessels (Figure 5B). Notably, more tiMDSCs (about 75%) were located in non-perfused areas (hypoxic/necrotic areas), compared to TAMs (about 50%) (Figure 5C). Consistently, immunohistochemistry data also showed that tiMDSCs were more located in distant to perfused vessels, while TAMs were relatively even distributed throughout the tumor tissue (Figure 5D). Thus, our data suggest that tiMDSC is more frequently located in hypoxic/necrotic areas.

### TiMDSC produces more pro-angiogenic factors than TAM

**Figure 6:**
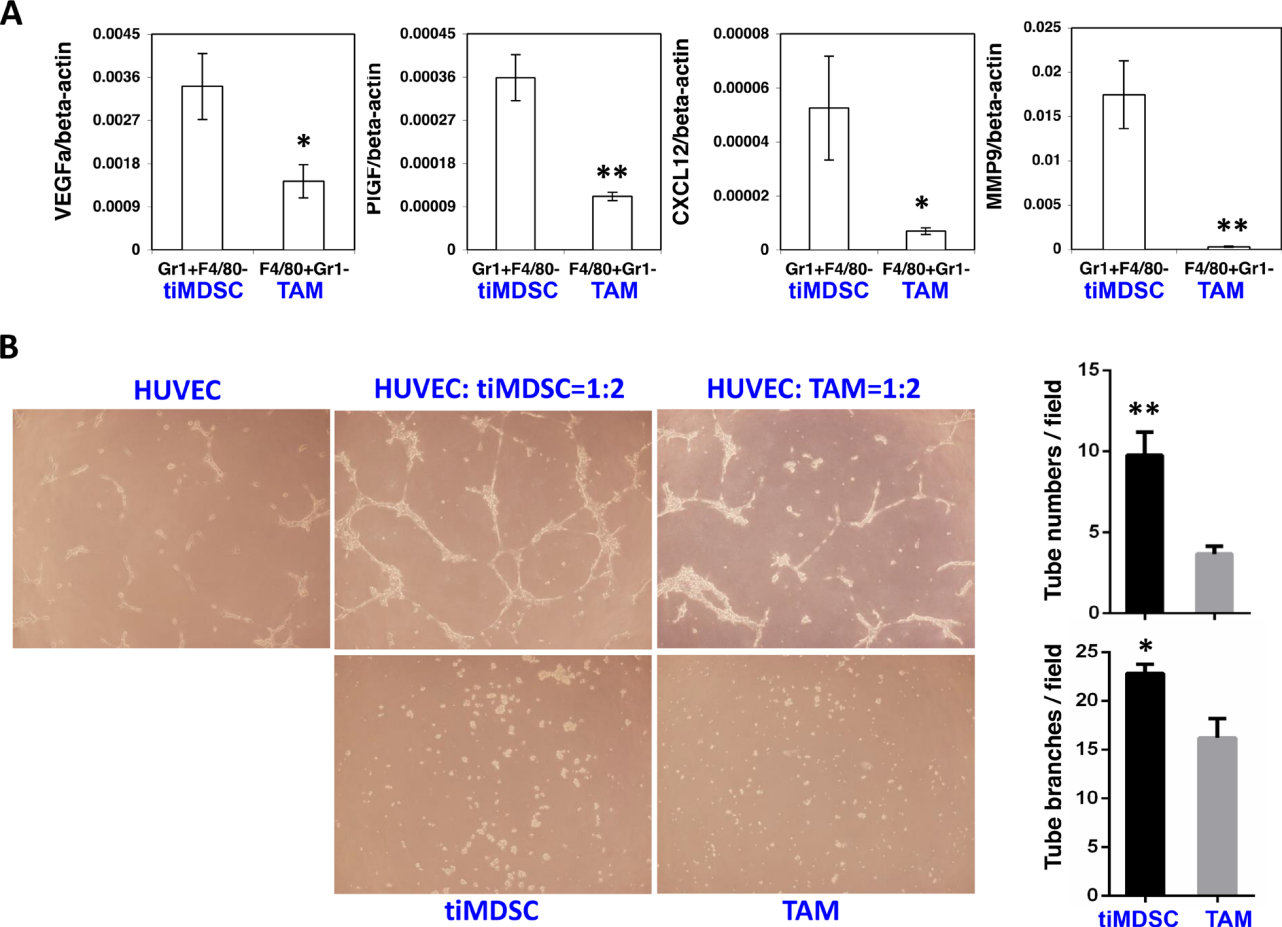
TiMDSC is more pro-angiogenic than TAM TiMDSC and TAM were flow sorted from MCaP0008 and MMTV-PyVT breast tumors as described in Figure 2. Gene expression of *VEGF, PlGF, CXCL12* and *MMP9* was analyzed by qPCR. (**A**) TiMDSC expressed higher levels of pro-angiogenic factors than TAM in MCaP0008 breast tumor. Experiments were repeated three times (*n* = 6 mice per group). (**B**) TiMDSC displayed stronger ability to promote tube formation, compared to TAM (*n* = 3 mice per group, MMTV-PyVT breast tumor model). Data were shown as mean values ± SEM.

Macrophage present in hypoxic areas displays altered gene expression with a pro-angiogenic phenotype [4, 27, 28]. Since tiMDSC is primarily present in non-perfused tumor areas, we proposed that tiMDSC and TAM possess differential pro-angiogenic capability. In flow sorted tiMDSCs and TAMs, tiMDSCs had significantly higher levels of *SDF1α*, *MMP9*, *VEGFa*, and *PlGF*, compared to TAMs (Figure 6A). Furthermore, in MMTV-PyVT breast tumors, tiMDSCs were more potent than TAMs in their ability to induce tube formation (Figure 6B). These data suggest that, in cell base, tiMDSC is more angiogenic than TAM.

### Breast tumor initiation is accompanied with a shift of tiMDSC to TAM

**Figure 7:**
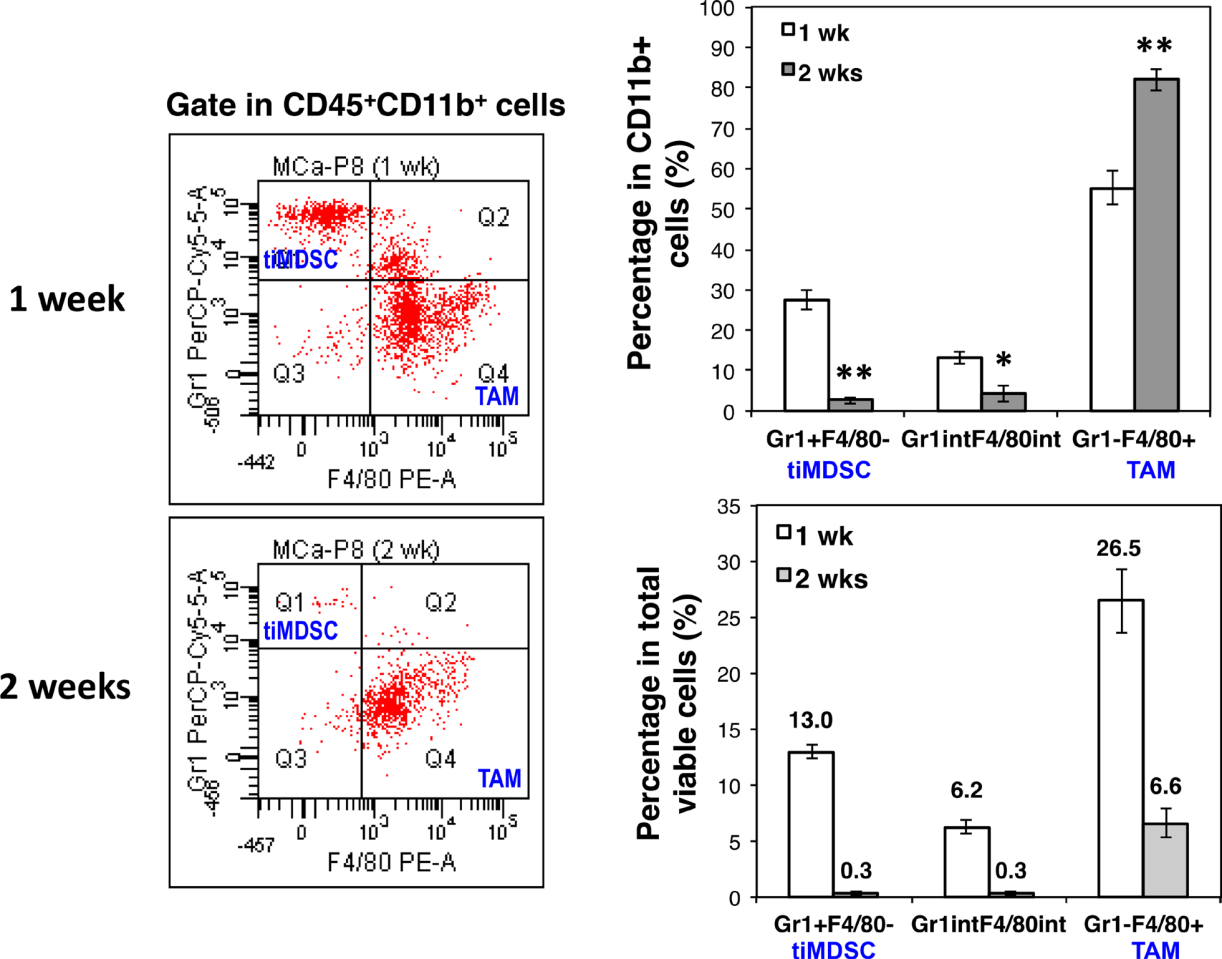
Breast cancer initiation is accompanied with a shift of tiMDSC to TAM MCaP0008 tumor cells (1 × 10^6^ cells) were inoculated in the mammary fat pads of FVB mice. Small tumors were harvested on day 7 or day 14 post tumor cell inoculation. Single cell suspensions were analyzed by flow cytometry. (**A**) Expression of Gr1 and F4/80 was analyzed in CD45^+^CD11b^+^ cells. The representative flow figures were shown. (**B**) The percentages of CD45^+^CD11b^+^Gr1^hi^F4/80^−^, CD45^+^CD11b^+^Gr1^int/low^F4/80^int/low^, and CD45^+^CD11b^+^Gr1^−^F4/80^+^ in total CD45^+^CD11b^+^ cells. Data were shown as mean values ± SEM (*n* = 6 mice per group). The experiment was repeated three times. * denotes *P* < 0.05, ** denotes *P* < 0.01.

The differentially immunosuppressive and angiogenic capabilities of tiMDSC and TAM (Figures 2 and 6) indicated that they may play different roles in tumor initiation and progression. Thus, we analyzed tiMDSC and TAM at two time points after MCaP0008 breast cancer cell inoculation. Interestingly, approximately 27.6% of the CD11b^+^ cells (13.0% in total viable cells) and 2.6% of total CD11b^+^ cells (0.3% in total viable cells) were tiMDSCs on day 7 and 14 after tumor cell inoculation, respectively (Figure 7). The absolute number of tiMDSC on day 7 and 14 was 4475 and 752 per tumor, and CD11b^+^ cells on day 7 and 14 was 16822 and 17812 per tumor. Conversely, 55.2% of CD11b^+^ cells (26.5% in total viable cells) were TAMs on day 7 post-inoculation, and that proportion increased to 82.0% (6.6% in total viable cells) on day 14 day after inoculation (Figure 7). The absolute number of TAM on day 7 and 14 was 9640 and 14913 per tumor, respectively. Although the percentages of tiMDSC and TAM decreased on day 14 relative to day 7, the ratios of TAM/tiMDSC were dramatically increased on day 14 (TAM/tiMDSC = 20) compared to day 7 (TAM/ tiMDSC = 2), suggesting a shift of tiMDSC to TAM. These data show that the initiation and progression of MCaP0008 breast tumors are accompanied by a shift of tiMDSC to TAM, which is consistent with their distinct angiogenic and immunosuppressive activities of tiMDSC and TAM.

### Spontaneous breast cancer lung colonization is associated with an increase in neutrophil

**Figure 8:**
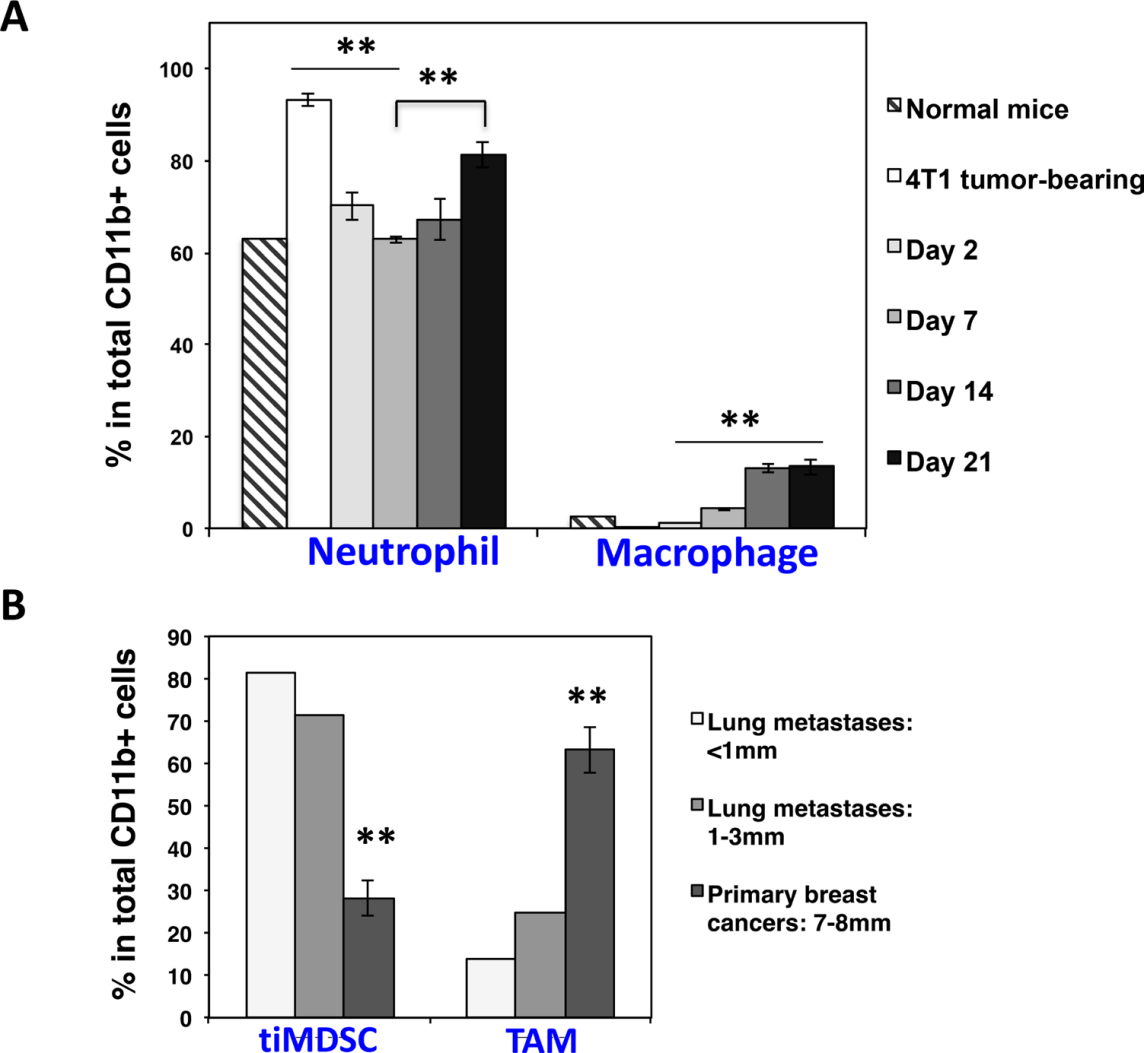
Spontaneous breast cancer lung colonization is associated with an increase of neutrophil in the lung 4T1 breast tumor cells were inoculated in Balb/c mice. Primary breast tumors were removed when tumors reached 5–7 mm in diameter. Neutrophil in the lung was analyzed by flow cytometry in different time points. (**A**) The proportion of neutrophil in the lung was decreased right after primary tumor removal and then rebounded during breast tumor lung colonization. Data were shown as mean values ± SEM (*n* = 3–5 mice per group). (**B**) The percentage of tiMDSC in lung metastases was higher than in primary breast cancers. Multiple different sizes of lung metastases from 3 lungs were dissected and pooled as two samples. Primary breast tumors: *n* = 5 mice per group. The experiment was repeated twice.

Breast cancer is the most common cancer in women. More than 90% of breast cancer-related deaths are due to metastasis of breast cancer cells into vital organs, especially the lung [31]. The colonization of metastatic cancer cells in distant organs is a key step for cancer progression. TiMDSC is preferentially accumulated during the early stage of breast cancer and possesses strong pro-angiogenic activity (Figures 6 and 7). Thus, we hypothesized that neutrophil facilitates lung colonization. To test this hypothesis, we analyzed the quantity of neutrophil in the lungs after we removed 4T1 primary tumors. Interestingly, the amount of neutrophil in the lungs dramatically decreased two days after primary tumor removal. On day 7, the levels of neutrophil were comparable to normal levels in the lung, and then began to increase (Figure 8A). We also examined lung metastases at different time points. After 4T1 primary tumor removal, there was no visible lung metastasis on day 2, and some of lungs had several tiny lung metastases on day 7. It is striking that there were dozens of lung metastases on day 14 and day 21. These data suggested that lung colonization by breast cancer cells was associated with an increase of neutrophil in the lung. We also compared tiMDSC present in different sizes of lung metastases. The proportion of tiMDSC in lung metastases was much higher than in that observed in primary breast tumors. In addition, the proportion of tiMDSC in small lung metastases was higher then larger lung metastases (Figure 8B). These data suggest that lung colonization by breast cancer cells is associated with the accumulation of neutrophil.

## DISCUSSION

Cancer immunotherapy and anti-angiogenic therapy are two key cancer treatment modalities [32, 33]. Tumor-infiltrating myeloid cells often compromise their efficacy [34, 35]. Myeloid cells are a heterogeneous cell population within the tumor immune microenvironment. They play critical roles in tumor angiogenesis and tumor immune evasion [34, 35], but the distinct characteristics of individual populations remain not fully defined. Here we showed that tiMDSC and TAM possess distinct angiogenic and immunologic properties in breast cancer models. In the established breast cancers, TAM is the most abundant immune cell population with potent immunosuppression. Therefore, polarization of TAM away from immunosuppression, or depletion of TAM could enhance cancer immunotherapy and/or improve survival [12, 23, 25, 36–38]. Conversely, tiMDSC is pro-angiogenic and preferentially accumulates during the early stage of breast cancer initiation or metastasis [14, 39]. Therefore, selectively targeting on distinct myeloid cell population based on breast cancer development stage could achieve better therapeutic outcome.

Tumor-infiltrating myeloid cells include multiple cell populations, including tiMDSC, TAM, TAN, granulocytic immature myeloid cell, and monocytic immature myeloid cell [5, 25, 35, 40]. Accumulating evidence suggests that there are phenotypic and functional overlaps between these populations. MDSC is initially identified in the peripheral immune organs. According to data on surface markers and their functions, MDSC within the tumor microenvironment may include neutrophil, monocyte and CD45^+^CD11b^+^Gr1^int/low^F4/80^low/+^ cell. Polymorphonuclear CD11b^+^Ly6G^+^Ly6C^low^ cells and monocytic CD11b^+^Ly6G^−^Ly6C^hi^ cells are two major populations in the spleen of tumor-bearing mice [9, 35, 41]. In MMTV-PyVT, tumor-infiltrating myeloid cells have two distinct populations based on the expression of Gr1 and F4/80: CD11b^+^Gr1^hi^F4/80^−^ and CD11b^+^Gr1^−^F4/80^+^. CD11b^+^Gr1^hi^F4/80^−^ cells, which are usually considered to be MDSC, are also Ly6G^+^Ly6C^low^, thus CD11b^+^Gr1^hi^F4/80^−^ MDSC phenotypically overlaps with TAN (CD11b^+^Ly6G^+^Ly6C^low^F4/80^−^) and granulocytic immature myeloid cell [5, 6, 25]. Since MDSC is generally considered to be a heterogeneous cell population, we suggest referring tumor-infiltrating CD11b^+^Ly6G^+^Ly6C^low^F4/80^−^ (tiMDSC) cell as TAN. Some investigators consider all tumor-infiltrating CD11b^+^ cells as MDSC, which includes both TAN and TAM [42, 43]. However, TAM is typically Gr1^−^F4/80^+^ and should not be considered as MDSC [4, 44]. Monocytic CD45^+^CD11b^+^Ly6G^−^Ly6C^+^F4/80^int/low^ cell population varies in different tumors and at different tumor stages, and it also changes by certain treatments [45, 46].

TAM is a multifunctional cell population. During tumor initiation, TAM forms an inflammatory environment to potentiate oncogenic mutations, but at the tumor progression stage, TAM secretes growth factors and cytokines to facilitate angiogenesis and suppress cytotoxic T cell responses. However, after progression to malignancy, TAM produces proteases and chemokines to induce tumor metastasis [37, 38, 47, 48]. Among myeloid cells present in breast cancers, TAM is very immunosuppressive. Interestingly, we found that tiMDSC was predominately located in less perfused and necrotic areas and expressed higher levels of pro-angiogenic factors. In addition, tiMDSC is more prevalent at early stages of cancer initiation or during metastatic cancer cell colonization. These properties suggest that tiMDSC is pro-angiogenic and pro-metastatic. This is consistent with previous reports that Gr1^+^ myeloid cells promote tumor angiogenesis and metastasis [14, 39, 49–51]. Gr1^+^ myeloid cells have been shown to be involved in the formation of lung metastatic niches [49].

Different myeloid cell populations possess various properties and may play different roles in tumor initiation and progression [9, 52, 53]. In general, the proportion of myeloid cells increases when tumor grows bigger in either peripheral immune organs or in the tumor microenvironment. Meantime, the composition of myeloid cell populations also changes at the various stages of tumor. We observed a higher ratio of tiMDSC/TAM during tumor initiation, compared to established tumors. TiMDSC is a heterogeneous cell population and can differentiate into other myeloid cell types. The shift of TAN to TAM could be due to the differentiation of tiMDSC to TAM. The hypoxic tumor microenvironment promotes rapid differentiation of tiMDSC into TAM [26, 54, 55]. In addition, the short lifespan of neutrophil may also contribute to the decreased ratio of tiMDSC/TAM [6].

Neutrophil is the first responder during inflammation. Indeed, CD11b^+^Gr1^+^ cells comprise about 75% of CD45^+^ cells infiltrated into inflammatory sites induced by OXd4 [56]. Tumor has been considered as a wound that does not heal. At the early stage of tumorigenesis, MDSC (CD11b^+^Gr1^+^) cells are the major infiltrating immune cells, which is likely due to inflammation [6]. During tumor development, the amount of TAM was increased and polarized to immunosuppression, and thus promoted tumor progression. TiMDSC appears to play a pivotal role in primary tumor initiation or metastatic cancer cell colonization via the induction of angiogenesis [13, 57], while TAM promotes tumor progression through the creation of an immunosuppressive tumor microenvironment. Thus, tiMDSC and TAM possess differential properties and fulfill distinct roles during different stages of cancer development and progression.

## MATERIALS AND METHODS

### Animals and tumor models

FVB/N, C57BL/6, Balb/c, and MMTV-PyVT mice were housed in pathogen free animal facilities. The EO771 breast tumor cell line was purchased from CH3 Biosystems (New York, USA). Dr. Peigen Huang at Massachusetts General Hospital generated the MCaP0008 breast tumor cell line [58]. Both of the cell lines were cultured in DMEM medium (Gibco, USA) containing 10% fetal bovine serum (FBS, Gibco). 4T1 breast tumor cells were purchased from ATCC (USA) and cultured in RPMI-1640 medium (Gibco) containing 10% FBS. To obtain source tumor tissue, MCaP0008 breast tumor cells (1 × 10^6^ cells) were injected orthotopically into the mammary fat pad of female FVB/N mice [58]. When the tumor reached 8 mm in diameter, it was excised, and a small piece (about 1 mm^3^) of viable tumor tissue was orthotopically transplanted into new female FVB mouse. In some experiments, both MCaP0008 (1 × 10^6^ cells) and EO771 (3 × 10^5^ cells) tumor cells were orthotopically inoculated into the mammary fat pads of FVB/N and C57BL/6 mice, respectively. Animal procedures were carried out following the Public Health Service Policy on Humane Care of Laboratory Animals. All procedures were approved by the Institutional Laboratory Animal Care and Use Committee of Soochow University.

### *In vivo* hoechst 33342 staining

When tumors reached 6−8 mm in diameter, tumor-bearing mice were injected, via the tail vein, with 200 μl of 10 mg/kg Hoechst 33342 dye (Sigma-Aldrich, USA). After 5 minutes, mice were systemically perfused with PBS and the tumors were removed. Under these conditions, a reproducible perivascular tumor cell-labeling gradient was achieved, as described previously [29, 30]. Tumor tissues were cut into two pieces. One portion was fixed with 4% paraformaldehyde for histological analysis. The remaining piece was used for flow sorting or flow cytometric analysis. FITC-anti-Ly6G (BD Biosciences, USA) and APC-anti-F4/80 (eBioscience, USA) were used to stained neutrophil and macrophage in the frozen tumor tissues.

### Isolation of tiMDSC and TAM

Tumors were harvested from MMTV-PyVT transgenic mice or MCaP0008 tumor-bearing mice. Tumor tissues were then minced and digested at 37°C for 45 min with DMEM containing collagenase type 1A (1.5 mg/ml), hyaluronidase (1.5 mg/ml), and DNase (20 U/ml). TiMDSCs and TAMs were enriched by CD11b-microbeads (Miltenyi, Germany). Enriched cells were then stained with PE-F4/80 (eBioscience), FITC-Ly6G, PE-Cy7-CD45, APC-Gr1, APC-Cy7-CD11b (BD Biosciences) and isolated by flow sorting. 50,000 tiMDSCs were subjected to cytospin preparation (Thermo Shandon), and were fixed and stained with a Wright Giemsa Staining according to the manufacturer's instructions (Sigma-Aldrich).

### Immune suppression assays

Mixed leukocyte reaction (MLR) was used to evaluate the immunosuppression of myeloid cell populations as previously described with minor modifications [9, 59]. Briefly, single cell suspensions of splenocytes were prepared from naïve mice. Splenocytes (2 × 10^5^ cells) were co-cultured in triplicate with tiMDSCs or TAMs (5 × 10^4^ cells) in a total volume of 200 ul containing anti-CD3/CD28 (1/5 μg/ml) for 24 hrs in U-bottom 96-well plates. Then the cultures were treated with 1 μCi of ^3^H-thymidine (GE Healthcare Life Sciences, USA) overnight. Cells were harvested using a cell harvester (Skatron Instruments), and ^3^H-thymidine uptaken was counted using a liquid scintillation counter. The background proliferation of splenocytes, tiMDSCs, or TAMs alone was subtracted to obtain the final proliferation value.

### Tube formation assays

The pro-angiogenic effect of myeloid cell populations was assessed by tube formation assay as descried previously with minor modifications [60, 61]. Briefly, human umbilical vein endothelial cells (HUVECs) were cultured in DMEM medium containing 10%FBS. Growth factor reduced matrigel matrix (CORNING) was thawed in a refrigerator (4°C) overnight. Matrigel matrix (100 μl/well) was added to the growth surface of 24-well plates and the coated plates were incubated at 37°C for 30 minutes to allow the gel to solidify. HUVECs (30,000 cells/well) were seeded onto the top of the gel and tiMDSCs or TAMs (60,000 cells/well) were added, and co-cultured with HUVECs in triplicate. Cells were incubated at 37°C with 5% CO_2_ for 12 hrs. The tube formation pictures were captured with a Box-Type Fluorescence Imaging Device (OLYMPUS). The numbers of tubes and branches were counted for at least three fields per well.

### Flow cytometric analysis

After intracardiac injection of PBS, breast cancer tissues, lung metastases or lungs were harvested, minced and digested at 37°C for 45 min with DMEM medium containing collagenase type 1A (1.5 mg/ml), hyaluronidase (1.5 mg/ml), and DNase (20 U/ml). The digestion mixtures were filtered through 70 μm cell strainers. Single-cell suspensions were incubated with rat anti-mouse CD16/CD32 mAb (BD Biosciences), and then stained, washed and re-suspended in cold buffer (1%BSA, 0.1% NaN3 in PBS). 7AAD reagent (eBioscience) was added to the stained tubes (5 μl/tube) just before running the flow analysis. Flow cytometry data were acquired on a Gallios flow cytometer (Beckman, USA), and data were analyzed with Kaluza software (version 1.3). The appropriate, fluorochrome-conjugated, isotype-matched, control IgGs were used in all experiments. The following monoclonal anti-mouse antibodies were used: CD45-PE-Cy7, CD45-PerCP, CD45-BV421, Gr1-PerCP-Cy5.5, Gr1-APC, Gr1-APC-Cy7, CD11b-APC-Cy7, CD11b-BV510, Ly-6G-FITC, Ly-6C-PE (BD Biosciences) and F4/80-PE, F4/80-FITC, F4/80-APC (eBioscience).

### Quantitative RT-PCR

Total RNA was extracted from the following flow-sorted cells using a RNeasy Mini Kit (QIAGEN, USA): tiMDSC, CD45^+^CD11b^+^Gr1^int/low^F4/80^int/low^, TAM. Full-length cDNAs were synthesized using an iScript™ cDNA Synthesis Kit (Bio-Rad, USA). Primers specific for β-actin, Arignase-1, IL10, CCL17, CCL22, MRC1, Nos2, IL12a, IL1β, TNFα, CXCL9, CXCL10, VEGF, *PlGF, CXCL12* and *MMP9* were provided in the Supplementary Table 1. qRT-PCR analysis was performed by using a Power SYBR^®^ Green PCR Master Mix (Applied Biosystems) on a 7500 Fast Real-Time PCR System (Applied Biosystems) according to the manufacturer's instructions. The comparative threshold cycle method was used to calculate fold change in gene expression, which was normalized to β-actin as a reference gene.

### Statistical analysis

Results were expressed as means ± SEM (Standard Error of Mean). Experimental differences were tested using Student's *t*-test (unpaired, two sided). Values were considered statistically significant when *P* < 0.05. Significant differences and the *p*-values are represented in figures by asterisks as follows: * < 0.05; ** < 0.01.

## SUPPLEMENTARY MATERIALS FIGURES AND TABLES



## Abbreviations

TAM: tumor-associated macrophage
MDSC: Myeloid-derived suppressor cell
tiMDSC: Tumor-infiltrating MDSC
TAN: tumor-associated neutrophil
SDF-1α: Stromal cell-derived factor-1α
MMP9: Matrix metallopeptidase 9
VEGFR2: Vascular endothelial growth factor receptor 2
VEGFa: Vascular endothelial growth factor a
PlGF: placenta growth factor
IL1β: Interleukin-1β
IL10: Interleukin-10
Arg1: Arginase 1
IL12a: Interleukin-12a
CXCL9: Chemokine (C-X-C motif) ligand 9
CXCL10: Chemokine (C-X-C motif) ligand 10
CCL17: Chemokine (C-C motif) ligand 17 and CCL22: Chemokine (C-C motif) ligand 22.
