# Supplementary material for: Myeloid-derived suppressor cell and macrophage exert distinct angiogenic and immunosuppressive effects in breast cancer
Source: Oncotarget. 2017 Apr 10;8(33):54173–86. doi: 10.18632/oncotarget.17013 (PMC5589571; doi:10.18632/oncotarget.17013)
Supplement: Supplementary file 1 [file oncotarget-08-54173-s001.pdf]

Myeloid-derived suppressor cell and macrophage exert distinct angiogenic and immunosuppressive effects in breast cancer

Supplementary Materials

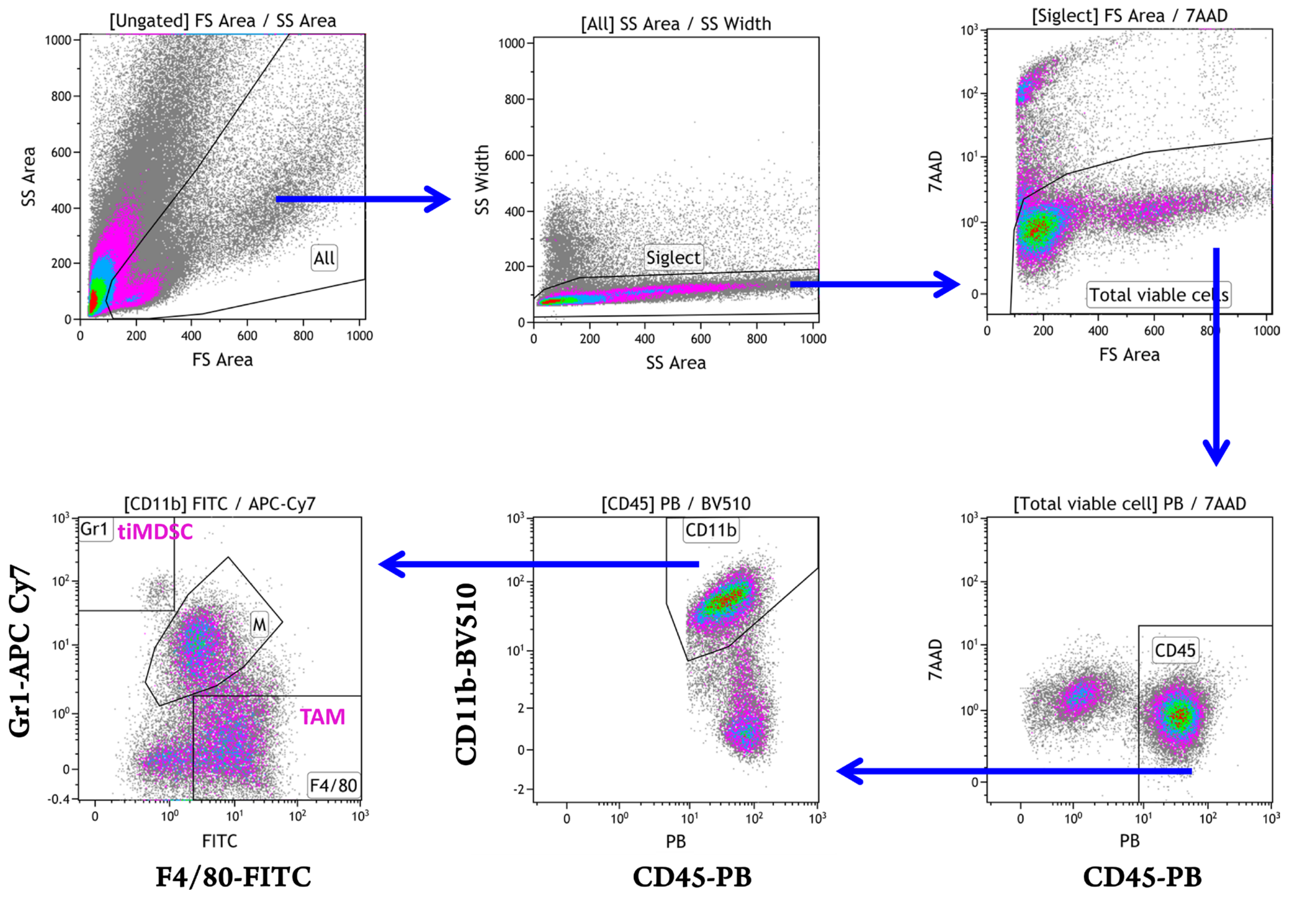

Supplementary Figure 1: The gating strategies of multicolor flow cytometric analysis to determine tumor-infiltrating myeloid cell populations in spontaneous MMTV-PyVT breast tumor tissues.

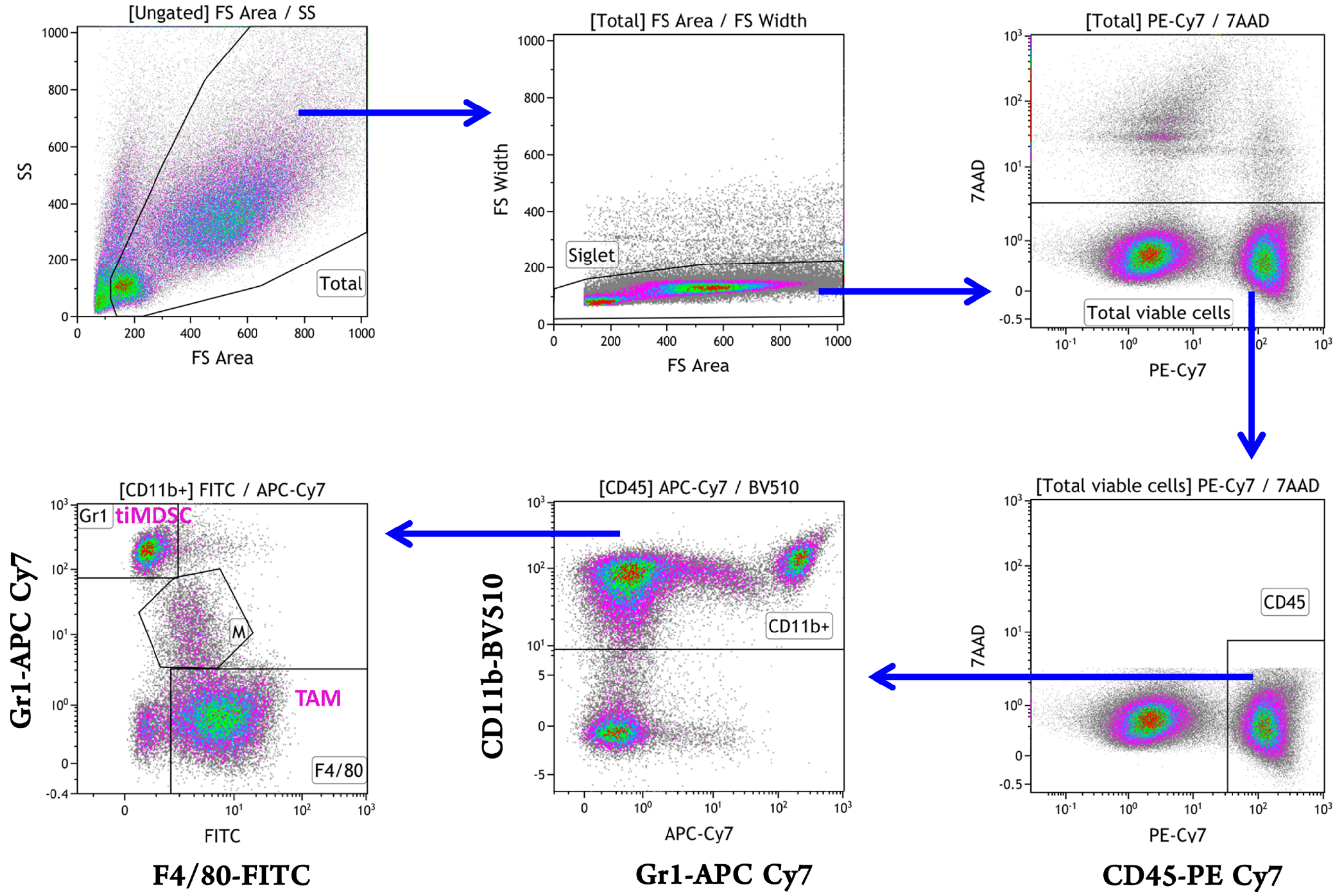

Supplementary Figure 2: The gating strategies of multicolor flow cytometric analysis to determine tumor-infiltrating myeloid cell populations in orthotopically implanted MCaP0008 breast tumor tissues.

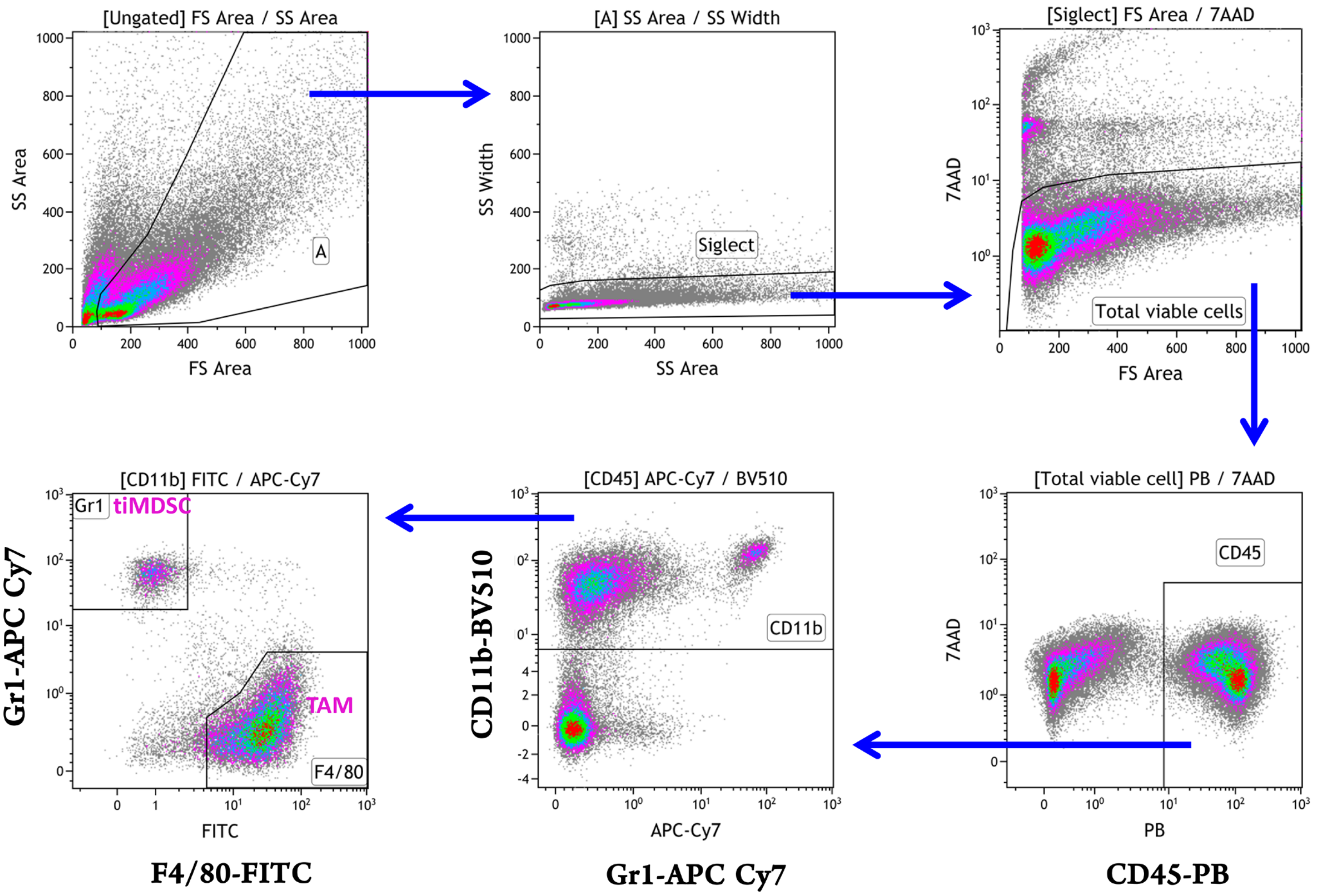

Supplementary Figure 3: The gating strategies of multicolor flow cytometric analysis to determine tumor-infiltrating myeloid cell populations in orthotopically inoculated EO771 breast tumor tissues.

Supplementary Table 1: Primers used for Q-PCR analysis

Gene	Primer	Sequence (5'-3')
β-actin	Forward<br>Reverse	ATCGTGCGTGACATCAAAGA<br>ACAGGATTCCATACCCAAGAAG
CCL17	Forward<br>Reverse	TGCTTCTGGGGACTTTTCTG<br>TGGCCTTCTTCACATGTTTG
CCL22	Forward<br>Reverse	GTCCTTCTTGCTGTGGCAAT<br>ACGGTTATCAAAACAACGCC
Arginase 1	Forward<br>Reverse	CAACCAGCTCTGGGAATCTG<br>AATCGGCCTTTTCTTCCTTC
IL10	Forward<br>Reverse	CCAGAGCCACATGCTCCTA<br>AGGGGAGAAATCGATGACAG
MRC1	Forward<br>Reverse	CCTGAACAGCAACTTGACCA<br>GCAATGGCCATAGAAAGGAA
TNFα	Forward<br>Reverse	CCGATGGGTTGTACCTTG TC<br>CGGACTCCGCAAAGTCTAAG
IL1β	Forward<br>Reverse	TGCCACCTTTTGACAGTGAT<br>TGTCCTCATCCTGGAAGGTC
IL12a	Forward<br>Reverse	GCCAGGTGTCTTAGCCAGTC<br>AGCTCCCTCTTGTTGTGGAA
IFNγ	Forward<br>Reverse	CCAAGTTTGAGGTCAACAACCC<br>GGGACAATCTCTTCCCCACC
iNOS	Forward<br>Reverse	CCACCTCTATCAGGAAGAAA<br>CTGCACCGAAGATATCTTCA
CXCL9	Forward<br>Reverse	AGTGTGGAGTTCGAGGAACC<br>GAGTCCGGATCTAGGCAGG
CXCL10	Forward<br>Reverse	TCATCCTGCTGGGTCTGAGT<br>CATCGTGGCAATGATCTCAACA
VEGFa	Forward<br>Reverse	CAGACAGTGCTCCAGCCG<br>CTGGGACCACTTGGCATGG
PlGF	Forward<br>Reverse	ATTCAGTCCGTCCTGTGTCC<br>GGTTTCCTCCTTTCTGCCT
CXCL12	Forward<br>Reverse	ACACTCCAAACTGTGCCCTT<br>TGAGCCTCTTGTTTAAAGCTTTCTC
MMP9	Forward<br>Reverse	CGTGTCTGGAGATTCGACTTGA<br>TTGGAAACTCACACGCCAGA
